# Polymorphism of DNA methyltransferase 3B −149C/T and cancer risk: a meta-analysis

**DOI:** 10.1007/s12032-014-0399-4

**Published:** 2014-11-30

**Authors:** Jing Zhu, Songtao Du, Jiaqi Zhang, Yingnan Wang, Qiaoling Wu, Jixiang Ni

**Affiliations:** 1 Department of Respiratory and Critical Care Medicine, The First People’s Hospital of Yichang, 2# Jiefang Road, Yichang, 443000 Hubei Province China; 2 Department of Respiratory and Critical Care Medicine, Renmin Hospital of Three Gorges University, Yichang, 443000 China

**Keywords:** DNMT3B, Polymorphism, Cancer, Meta-analysis

## Abstract

Published data on the association between DNA methyltransferase (DNMT) 3B −149C/T polymorphism and cancer risk remain inconclusive. To derive a more precise estimation for this association, we performed a meta-analysis of 5,903 cancer cases and 8,132 controls from 22 published case–control studies. We used odds ratios (ORs) with 95 % confidence intervals (CIs) to assess the strength of the association. Our meta-analysis suggested that DNMT3B −149C/T polymorphism was associated with the risk of head and neck cancer under heterozygote comparison (OR 0.73, 95 % CI 0.59–0.90) and dominant model (OR 1.75, 95 % CI 0.62–0.92), although no evidence of association between DNMT3B −149C/T polymorphism and cancer risk was observed as we compared in the pooled analyses (homozygote comparison: OR 0.96, 95 % CI 0.86–1.09; heterozygote comparison: OR 1.07, 95 % CI 0.86–0.32; dominant model: OR 1.03, 95 % CI 0.85–1.25; recessive model: OR 0.93, 95 % CI 0.8–1.08). More studies are needed to detect DNMT3B −149C/T polymorphism and its association with cancer in different ethnic populations incorporated with environment exposures in the susceptibility of different kinds of cancer.

## Introduction

DNA methylation is a major epigenetic modification that involves the addition of a methyl group to the 5′ position of a cytosine in a CpG dinucleotide, which is catalyzed by a family of DNA methyltransferases (DNMTs) including three activated forms (DNMT1, DNMT3A and DNMT3B) in human [1, 2]. Aberrant DNA methylation is one of the most consistent epigenetic changes observed in human cancers [3]. DNMT1 is a maintenance DNA methyltransferase, whereas DNMT3A and DNMT3B are considered as de novo methyltransferases because they can methylate unmethylated and hemi-methylated DNA with equal efficiency [4, 5]. A number of studies showed that DNMT3B was up-regulated in several human cancers, demonstrating that DNMT3B may play an important role in tumorigenesis by contributing to the generation of aberrant DNA methylation [6–8].

The DNMT3B gene is assigned to chromosome 20q11.2 and contains a single C→T transition polymorphism (C46359T) at a novel promoter region, −149 base pairs from the transcription start site, which may result in greatly increased promoter activity of the gene [9]. A number of single-nucleotide polymorphisms (SNP) in the gene have been described in the literature, of which a common SNP −149C/T (rs2424913) in the promoter region of the DNMT3B is known to regulate its expression [10]. Recently, a variety of molecular epidemiological studies have been conducted to examine the association between DNMT3B −149C/T polymorphism and cancer susceptibility [11–31], but the results remain inconclusive. Therefore, the association between DNMT3B −149C/T polymorphism and cancer risk requires further investigation.

Considering the relatively small sample size in most studies, it is possible to perform a quantitative synthesis of the evidence with rigorous methods. Here, we performed a meta-analysis on 22 published case–controls to derive a more precise evaluation of the association between DNMT3B −149C/T polymorphism and cancer risk.

## Materials and methods

### Identification and eligibility of relevant studies

A systematic literature search was performed using PubMed, Medline, EMBASE and Chinese National Knowledge Infrastructure (CNKI), covering all articles published up to October 2014. We used the following terms: “DNMT3B,” “polymorphism,” “rs2424913” and “cancer”. References of the retrieved publications were also screened. All eligible studies were retrieved, and their bibliographies were checked for other relevant publications. Only published studies with full-text articles were included. When overlapping articles were found, we only included the publications that reported the most extensive information.

### Inclusion criteria

The inclusion criteria were as follows: (1) published in English or in Chinese; (2) case–control studies of cancer with DNMT3B −149C/T polymorphism; (3) supply the available genotype frequencies in cancer cases and controls; and (4) sufficient published data for estimating an odds ratio (OR) with 95 % confidence interval (CI).

### Data extraction

Two investigators independently (Jing Zhu and Songtao Du) reviewed the articles to exclude irrelevant and overlapping studies. The results were compared, and disagreements were resolved by discussion and consensus. We extracted the following information from each study: first author’s surname, year, ethnicity, tumor type, definition of cases, characteristics of controls, validity of the genotyping method, and the number of cases and controls for each genotype.

### Statistical analysis

OR and 95 % CI were used to assess the strength of association between DNMT3B −149C/T polymorphism and the risk of cancer under homozygote comparison (CC vs. TT), heterozygote comparison (CT vs. TT), dominant (CC/CT vs. TT) and recessive (CC vs. CT/TT) genetic model comparison. The significance of the combined OR was determined by the *Z* test, in which *P* < 0.05 was considered significant. Stratified analyses were also performed by cancer types, ethnicities, and sources of controls. The Chi-square-based Q statistic test was performed to evaluate the between-study heterogeneity of studies. If *P* < 0.1, between-study heterogeneity was considered to be significant [32]. When the effects were assumed to be homogenous, the fixed effects model based on Peto method was used, otherwise, the random effects model based on Mantel–Haenszel method was applied. We also used the statistic of *I* ^2^ to efficiently test for the heterogeneity, with *I* ^2^ < 25 %, 25–75 % and >75 % to represent low, moderate and high degree of inconsistency, respectively [33]. Funnel plots were used to access the potential publication bias by the method of Egger’s linear regression test [34]. All analyses were performed by Stata (version 10.0, Stata Corporation) and Review Manager (version 5.0.0, The Cochrane collaboration), using two side *P* values.

## Results

### Characteristics of studies

Twenty two case–control studies including 5,903 cancer cases and 8,132 controls met the including criteria. The study characteristics were listed in Table 1. Most of cases in the studies were histologically diagnosed, and most of the controls were selected from healthy population. Fifteen studies used frequency-matched controls to the cases by age, sex, residence or ethnicity. A classic polymerase chain reaction–restriction fragment length polymorphism assay was performed in all studies (Table 1).

**Table 1:** Characteristics of published studies included in this meta-analysis

Authors	Year	Ethnicity	Tumor type	Definition of cases	Characteristics of controls (matched for)	Methods	Sample size
Bao [11]	2011	Asian	Colorectal cancer	Histologically confirmed	Healthy (age, gender, and residence)	PCR–RFLP	544/533
Fan [12]	2008	Asian	Colorectal cancer	Histologically confirmed	Healthy (age, gender, residence and ethnicity)	PCR–RFLP	137/308
Joes [13]	2006	Mixed	Colorectal cancer	Not described	Unclear (age, gender and ethnicity)	PCR–SSCP	74/72
Karpinski [14]	2010	Occident	Colorectal cancer	Not described	Healthy (age, gender, residence and ethnicity)	PCR–RFLP	186/140
de Vogel [15]	2009	Occident	Colorectal cancer	Histologically confirmed	Healthy (age, gender)	PCR–RFLP	703/1,810
Iacopetta [16]	2009	Occident	Colorectal cancer	Histologically confirmed	Healthy (age, gender, and residence)	PCR–RFLP	828/949
Reeves [17]	2008	Occident	Colorectal cancer	Not described	Healthy (age, gender, and ethnicity)	PCR–RFLP	194/210
Aung [18]	2005	Asian	Gastric cancer	Histologically confirmed	Healthy (age, gender)	PCR–RFLP	152/247
Hu [19]	2010	Asian	Gastric cancer	Histologically confirmed	Healthy (age, gender, and residence)	PCR–RFLP	259/262
Wang [20]	2005	Asian	Gastric cancer	Histologically confirmed	Healthy (age, gender, and residence)	PCR–RFLP	212/294
Succi [21]	2013	Occident	HNSCC	Histologically confirmed	Healthy (gender)	PCR–RFLP	237/488
Liu [22]	2008	Occident	HNSCC	Histologically confirmed	Healthy (age, gender)	PCR–RFLP	832/843
Ezzikouri [23]	2009	African	Hepatocellular carcinoma	Not described	Unclear (age, gender and ethnicity)	PCR–RFLP	96/222
Wu [24]	2007	Asian	Hepatocellular carcinoma	Histologically confirmed	Healthy (age, gender and ethnicity)	PCR–RFLP	100/140
Lao [25]	2013	Asian	Hepatocellular carcinoma	Not described	Healthy (age, gender)	PCR–RFLP	108/216
Eftekhar [26]	2014	Asian	Breast cancer	Histologically confirmed	Healthy (age)	PCR–RFLP	100/138
Montgomery [27]	2004	Occident	Breast cancer	Not described	Unclear (age)	PCR–RFLP	352/258
Li [28]	2005	Asian	Acute leukemia	Not described	Healthy	PCR–RFLP	160/240
Shen [10]	2002	Occident	Lung cancer	Histologically confirmed	Unclear (age, gender, residence and ethnicity)	PCR–RFLP	319/340
Singal [29]	2005	Occident	Prostate cancer	Not described	BPH	PCR–RFLP	81/42
Hernández-Sotelo [30]	2013	Occident	Cervical cancer	Histologically confirmed	Healthy (age)	PCR–RFLP	70/200
Mostowska [31]	2013	Occident	Ovarian cancer	Histologically confirmed	Healthy (age)	PCR–RFLP	159/180

*PCR* Polymerase chain reaction, *RFLP* restriction fragment length polymorphism, *HNSCC* head and neck squamous cell carcinoma, *BPH* benign prostatic hypertrophy

### Main results

**Fig. 1:**
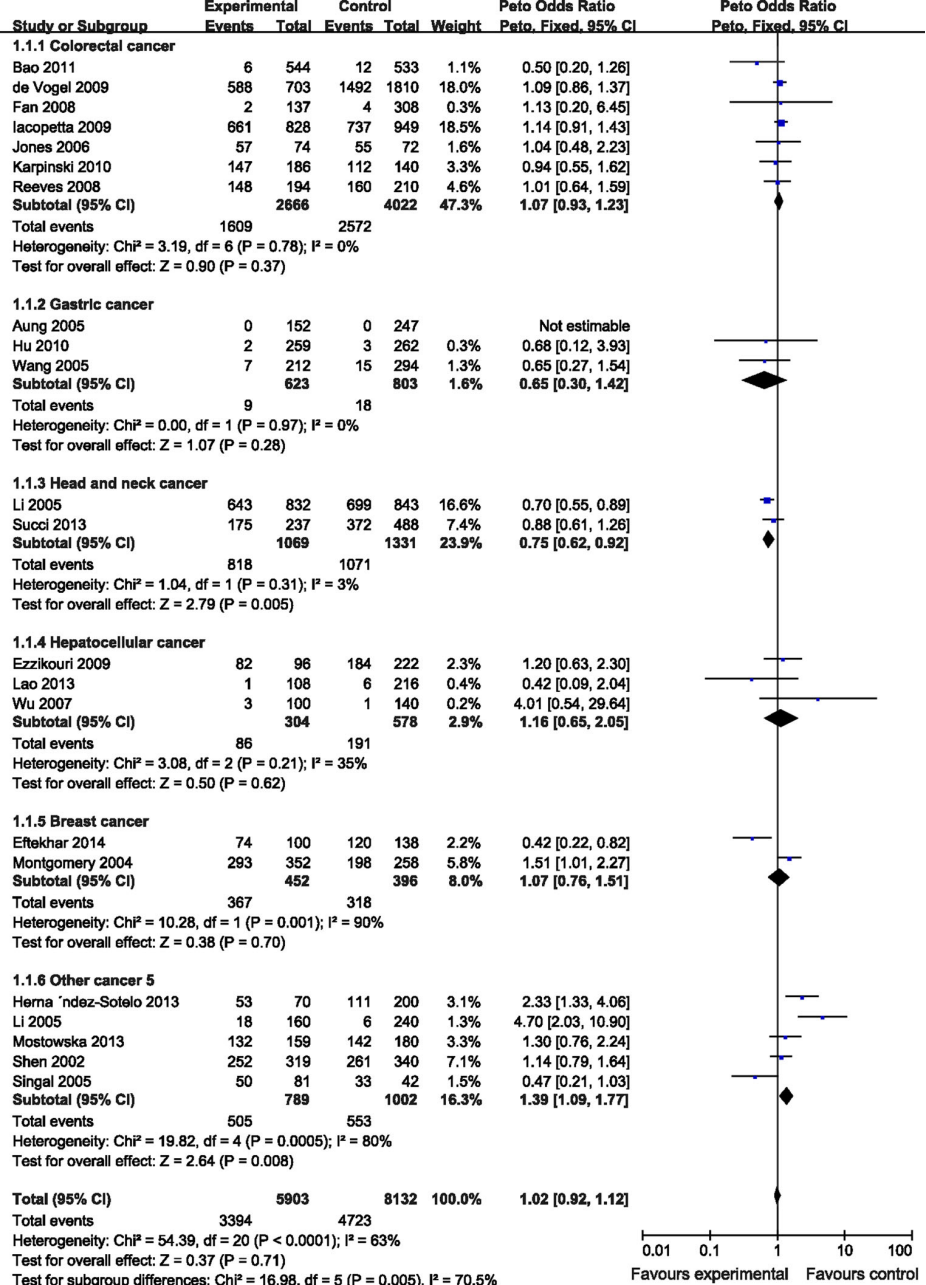
Meta-analysis with a fixed effects model for the ORs of cancer risk associated with DNMT3B −149 C/T (CC/CT vs. TT)

The evaluation of association between DNMT3B −149C/T polymorphism and cancer risk is presented in Table 2. There was no significant association between DNMT −149C/T polymorphism and the risk of cancer (CC vs. TT: OR 0.96, 95 % CI 0.86–1.09; *P* = 0.1, *I* ^2^ = 34 % for heterogeneity). In the stratified analysis by cancer type, DNMT3B −149C/T polymorphism was relative with a significantly increased risk of head and neck cancer in two tested models (CT vs. TT: OR 0.73, 95 % CI 0.59–0.9; *P* = 0.33, *I* ^2^ = 0 % for heterogeneity; CC/CT vs. TT: OR 0.76, 95 % CI 0.61–0.93; *P* = 0.3, *I* ^2^ = 7 % for heterogeneity; Fig. 1). However, no significant elevated risk of colorectal cancer, gastric cancer, hepatocellular cancer, breast cancer and other cancers with this polymorphism were shown in overall comparisons. At the same time, we failed to find significant main effects for DNMT3B −149C/T polymorphism on cancer risk in different genetic models when stratified according to ethnicity or sources of controls.

**Table 2:** Total and stratified analyses of the DNMT3B −149C/T polymorphism on cancer risk

Variable	No.^a^	Cases/controls	CC versus TT			CT versus TT			CC/CT versus TT			CC versus CT/TT		
			OR (95 % CI)	*P* ^b^	*P*	OR (95 % CI)	*P* ^b^	*P*	OR (95 % CI)	*P* ^b^	*P*	OR (95 % CI)	*P* ^b^	*P*
Total	22	5,903/8,132	0.96 [0.86, 1.09]	0.10	0.55	1.07 [0.86, 1.32]	0.00^c^	0.56	1.03 [0.85, 1.25]	0.00^c^	0.76	0.93 [0.80, 1.08]	0.01^c^	0.36
Ethnicities														
Occident	11	3,961/5,460	0.98 [0.87, 1.1]	0.11	0.72	1.10 [0.89, 1.37]	0.00^c^	0.38	1.06 [0.88, 1.28]	0.00^c^	0.54	0.95 [0.83, 1.09]	0.07^c^	0.45
Asian	9	1,772/2,378	0.78 [0.36, 1.7]	0.15	0.53	0.87 [0.40, 1.92]	0.00^c^	0.74	0.91 [0.42, 1.95]	0.00^c^	0.81	1.60 [0.87, 2.93]	0.31	0.13
African	1	96/222	1.16 [0.56, 2.39]	NE^d^	0.70	1.25 [0.56, 2.39]	NE^d^	0.54	1.21 [0.62, 2.35]	NE^d^	0.58	0.98 [0.59, 1.63]	NE^d^	0.94
Mixed	1	74/72	0.44 [0.17, 1.11]	NE^d^	0.53	1.67 [0.73, 3.80]	NE^d^	0.22	1.04 [0.48, 2.23]	NE^d^	0.93	0.32 [0.16, 0.67]	NE^d^	0.02
Cancer types														
Colorectal cancer	7	2,666/4,022	1.06 [0.9, 1.25]	0.37	0.48	1.08 [0.93, 1.26]	0.67	0.32	1.07 [0.93, 1.23]	0.78	0.37	0.93 [0.74, 1.17]	0.05^c^	0.53
Gastric cancer	3	623/803	NE^d^			1.65 [0.30, 1.42]	0.97	0.28	1.65 [0.30, 1.42]	0.97	0.28	NE^d^		
Head and neck cancer	2	1,069/1,331	0.80 [0.63, 1.01]	0.33	0.06	0.73 [0.59, 0.90]	0.34	0.003	1.75 [0.62, 0.92]	0.31	0.005	1.00 [0.84, 1.20]	0.67	0.98
Hepatocellular cancer	3	304/578	1.16 [0.56, 2.39]	NE^d^	0.70	1.18 [0.65, 2.14]	0.21	0.59	0.16 [0.65, 2.05]	0.21	0.62	0.98 [0.59, 1.63]	NE^d^	0.94
Breast cancer	2	452/396	0.80 [0.63, 1.01]	0.33	0.06	0.75 [0.18, 3.15]	0.00^c^	0.69	0.83 [0.24, 2.83]	0.00^c^	0.76	1.20 [0.89, 1.61]	0.37	0.24
Other cancers	5	789/1,002	1.23 [0.83, 1.83]	0.10	0.3	1.65 [0.92, 2.93]	0.04^c^	0.09	1.48 [0.82, 2.68]	0.00^c^	0.19	0.69 [0.42, 1.13]	0.09^c^	0.14
Sources of controls														
Hospital based	11	2,929/3,139	0.81 [0.53, 1.22]	0.03^c^	0.31	0.89 [0.63, 1.26]	0.03^c^	0.52	0.85 [0.63, 1.17]	0.06^c^	0.32	0.85 [0.62, 1.15]	0.05^c^	0.29
Population based	11	2,974/4,993	1.03 [0.89, 1.19]	0.51	0.68	1.19 [0.91, 1.56]	0.00^c^	0.21	0.15 [0.91, 1.45]	0.00^c^	0.24	0.96 [0.80, 1.16]	0.02^c^	0.69

0.00 means value <0.01

^a^Number of studies

^b^ *P* value of *Q* test for heterogeneity test

^c^Random effects model was used when *P* value for heterogeneity test <0.10; otherwise, fixed effects model was used

^d^Not estimable

### Test of heterogeneity

There was significant heterogeneity for recessive model comparison (CC vs. CT/TT: *P*_heterogeneity_ = 0.01), for heterozygote comparison (CT vs. TT: *P*_heterogeneity_ < 0.001) and for dominant model comparison (CC/CT vs. TT: *P*_heterogeneity_ < 0.001), but not for homozygote comparison and (CC/TT: *P*_heterogeneity_ = 0.1). Then, we assessed the source of heterogeneity for homozygote comparison by cancer type, ethnicity and source of controls. As a result, cancer type (*χ* ^2^ = 7.04, *df* = 4, *P* = 0.13), ethnicity (*χ* ^2^ = 3.36, *df* = 3, *P* = 0.34) and source of controls (*χ* ^2^ = 2.56, *df* = 1, *P* = 0.11) were not found to contribute to substantial heterogeneity.

### Sensitivity analysis

Sensitivity analysis was performed by sequential omission of individual studies in whole subjects and subgroups, respectively. For DNMT3B −149C/T, the significance of pooled ORs was influenced evidently by individual study on the whole population or subgroup analysis of cancer type and ethnicity. In the cancer type subgroup analysis, the study of Jones et al. [13] was the main originators of heterogeneity in the colorectal cancer. When the study was excluded, heterogeneity was significantly decreased (CC vs. CT/TT: *P*_heterogeneity_ = 0.94, *I* ^2^ = 0 %). Similarly, when study by Mostowska et al. [31] was excluded, heterogeneity was also decreased in other type cancer (CC vs. CT/TT: *P*_heterogeneity_ = 0.34, *I* ^2^ = 11 %). Additionally, in the ethnicity subgroup analysis, sensitivity analyses suggested that the study [28] was the main originator of heterogeneity in Asian. After exclusion of this study, heterogeneity was significantly decreased (CT vs. TT: Pheterogeneity = 0.37, *I* ^2^ = 8 %; CC/CT vs. TT: Pheterogeneity = 0.37, *I* ^2^ = 0 %).

### Publication bias

**Fig. 2:**
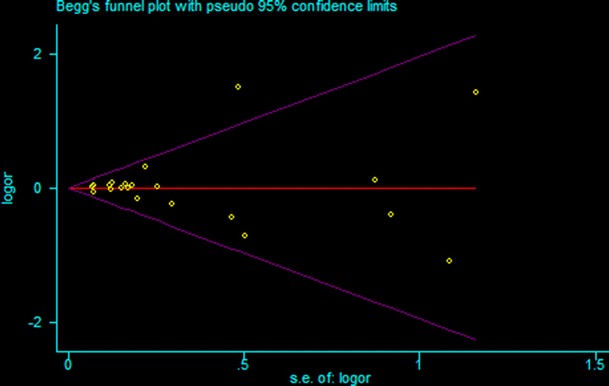
Funnel plot for publication bias of the meta-analysis of cancer risk and DNMT3B −149C/T polymorphism (CC/CT vs. TT)

Funnel plots are shown in Fig. 2 for dominant model. Arrangement of data points did not reveal any evidence of obvious asymmetry. Formal evaluation using Egger’s regression asymmetry tests for dominant model and the result still did not show any evidence of publication bias (*t* = 0.25, *P* = 0.80).

## Discussion

The present meta-analysis, including 5,903 cancer cases and 8,132 controls from 22 published case–control studies, showed that the DNMT3B −149C/T was not associated with cancer risk. When stratified by different types of cancer, we found an association between DNMT3B −149C/T polymorphism and head and neck cancer risk under heterozygote comparison and dominant model, but there are only two studies in analysis with limited sample size; therefore, the result should be interpreted with caution. Given the important roles of DNMT3B in cancer risk, it was biologically possible that DNMT3B polymorphism is associated with the risk of cancer by increasing DNMT3B promoter activity that modulated an aberrant de novo methylation of CpG islands in some tumor suppressor genes [4]. Studies on the functionality of this polymorphism might contribute to a better understanding of tumor biology and behavior and help us to predict the genetic susceptibility of cancer and choose therapies in an individual manner. However, DNMT3B −149C/T polymorphism did not increase the risk of colorectal cancer, gastric cancer, breast cancer and hepatocellular carcinoma in overall population. The probability may be that different types of cancer may have different mechanism of carcinogenesis. The differences in genetic background and/or environmental exposure may result in different frequency of −149 C/T genotype in healthy individuals from distinct ethnicities; however, in subgroup analysis by ethnicity, we also did not find that DNMT3B −149C/T was associated with ethnicity. It is likely that the small sample size may have insufficient statistical power to detect a real effect. Therefore, more studies based on large population and more different ethnicity should be conducted to further examine this association.

Heterogeneity is a potential problem when interpreting the results of all meta-analysis. Although we minimized the likelihood by performing a careful search for published studies, using strict criteria for study inclusion, precise data extraction and careful data analysis, significant between-study heterogeneity existed in most comparisons. After subgroup analysis by cancer types, ethnicity and source of controls, the heterogeneity was effectively decreased, but significant heterogeneity still existed. Thus, we choose to use random effects model, when *I* ^2^ value for heterogeneity test is <50 %. The reason might be that different genetic backgrounds and the environment existed among different ethnicities and individuals.

Numbers of SNPs, however, were frequently investigated in the former studies to evaluate the association between DNMT3B polymorphisms and cancer in diverse populations. There might be some other SNPs in DNMT3B associated with risk of cancer. Lee et al. [35] found C alleles of DNMT3B contributed to the susceptibility of lung cancer in Korean population. Some other SNPs of DNMT3B, such as −579 G/T and −283 T/C, were also researched by some studies on their association with cancer risk [11, 12, 14, 19, 36, 37]. However, there were only a very limited number of studies available for some SNPs and therefore not having enough statistical power to explore the real association.

Some other limitations in our meta-analysis should be acknowledged. Firstly, controls were not uniformly defined, while our result was based on unadjusted estimates. Secondly, in the subgroup analyses, the sample size of different types of cancer was relatively small, such as lung cancer, ovarian cancer and prostate cancer not having enough statistical power to explore the real association. Thirdly, only English and Chinese language studies were included in this meta-analysis might have led to publication bias, and the exclusion of unpublished data was generally associated with an overestimation of the true effect.

In conclusion, our meta-analysis suggested that DNMT3B −149C/T polymorphism was not related to overall cancer risk, whereas there was an association between DNMT3B −149C/T polymorphism and head and neck cancer risk under heterozygote comparison and dominant model. Larger samples among different populations, especially more sophisticated gene–gene and gene–environment interactions should be considered in future studies, which should lead to better, comprehensive understanding of the association between DNMT3B −149C/T polymorphism and cancer risk.
